# *Streptococcus pneumoniae* as a cause of lactational mastitis: a case report

**DOI:** 10.1002/ccr3.1488

**Published:** 2018-03-25

**Authors:** Sara Vester Hald, Henrik Carl Schønheyder

**Affiliations:** ^1^ Department of Gynecology and Obstetrics Aalborg University Hospital Aalborg Denmark; ^2^ Department of Clinical Microbiology Aalborg University Hospital Aalborg Denmark; ^3^ Department of Clinical Medicine Aalborg University Aalborg Denmark

**Keywords:** Mastitis, pneumococcal bacteremia, pneumococcal infection, puerperal mastitis, *Streptococcus pneumoniae*

## Abstract

Pneumococci are predominant in respiratory tract infections but may have unusual presentations such as lactational mastitis. We report a 29‐year‐old breastfeeding woman with pneumococcal mastitis confirmed by positive blood culture and culture of milk. Previously, a few similar cases have been reported, but none was associated with bacteremia.

## Introduction

Pneumococci are worldwide frequent causes of pneumonia, otitis media, bacterial meningitis, and sepsis, and this may obscure the fact that pneumococci have an even more diverse clinical spectrum. We report a 29‐year‐old breastfeeding woman with lactational mastitis three months post partum. The pneumococcal etiology was confirmed by cultures of blood and milk. Only few cases of pneumococcal mastitis have been reported, and our patient was the first with documented bacteremia. The rarity concurs with a low reported prevalence of pneumococci in samples of milk obtained for diagnosis or milk bank quality control.

Among breastfeeding women, the incidence of mastitis varies from 2.6% to 33% 1. It may occur at every stage of lactation, but 74–95% occur during the first twelve weeks post partum 1.

Duct obstruction and a continuum of milk stasis play a key role in the pathogenesis of puerperal mastitis, and the role of bacteria is less clearly defined. *Staphylococcus aureus* is the microorganism most frequently associated with infectious mastitis followed by coagulase‐negative staphylococci (CoNS), group B streptococci, viridans streptococci, and *Enterococcus faecalis* 2.

We present a case of puerperal mastitis with the rare finding of *Streptococcus pneumoniae* in both milk culture and blood culture.

## Case Report

A healthy 29‐year‐old woman was admitted with pyrexia and tenderness of the right breast 3 month after giving birth to her second child. Implants for breast augmentation had been inserted 8 years previously without complications. The lateral side of her right breast had been red and tender 2 weeks earlier, and her general practitioner had prescribed pivampicillin orally with relief of symptoms in response. However, the symptoms recurred in the medial part of the right breast 2 days before the admission.

At admission, the patient appeared septic; presenting with hypotension (systolic blood pressure at 89 mmHg), a high fever (39.7˚C), tachycardia (130 beats per minute), and tachypnea (respiratory rate at 22). The primary physical examination revealed an indurated and tender right breast with a small area of erythema on the medial side. There was purulent secretion from the nipple but no axillary adenopathy nor fluctuation in the breast. A sample of milk was obtained for culture as well as a blood culture (BACTEC, Becton‐Dickinson, Franklin Lakes, NJ).

Laboratory investigations showed an elevated total leucocyte count at 14.9 × 10^9^ cells/L (normal range 3.5–10 × 10^9^ cells/L) and C‐reactive protein 76 mg/L (normal range <10 mg/L). Ultrasound examination excluded abscess formation and confirmed breast augmentation surgery.

Oral dicloxacillin was instituted but the patient remained febrile, and swelling of the breast and erythema expanded. Breastfeeding the baby failed, although the patient continued pumping the breastmilk.

On day two, C‐reactive protein had increased to 283 mg/L. *S. pneumoniae* was reported both from the blood culture and the milk sample. The isolates were confirmed to be penicillin susceptible by screening with an oxacillin 1 *μ*g disk, and the antibiotic therapy was switched to intravenous benzylpenicillin with a rapid clinical response.

On the third day of admission, the patient was afebrile and there was a marked relief of symptoms from the breast. A second ultrasound examination showed edema of the gland tissue and no abscess. The total leucocyte count was 7.0 × 10^9^ cells/L and the C‐reactive protein had also decreased to 119 mg/L. The patient was discharged on the third day with a 7‐day course of phenoxymethylpenicillin. Two weeks postdischarge, a third ultrasound examination showed normal configuration of the breast tissue. The patient had resumed breastfeeding from both breasts.

The baby was a healthy companion throughout the admission, and no nasopharyngeal swab was performed.

The blood culture isolate was later reported to be serotype 19F.

## Discussion

We have identified four previously published cases of pneumococcal mastitis in breastfeeding women spanning a 20‐year period (Table 1). Including our case, the median time before infection was 6 months (range 3–9 months). The serotypes reported are common among pneumococci colonizing the nasopharynx during infancy and are compatible with transmission from the baby's nasopharynx during breastfeeding. We refrained from obtaining a nasopharyngeal swab because the baby was healthy.

**Table 1:** Key features of the current case of pneumococcal lactational mastitis and four previously reported cases

Author	Age of patient (years)	Time from birth (months)	Localization of mastitis	Milk culture results	Blood culture results	Nasopharyngeal culture from the child	Treatment
Present case	29	3	Right medial breast	*S. pneumoniae*	*S. pneumoniae* serotype 19F	n.d.	Dicloxacillin → Benzylpenicillin → Phenoxymethylpenicillin
Wüst et al., 1995 5	38	9	Left medial breast	*S. pneumoniae* serotype 6B	n.a.	*S. pneumoniae* serotype 6B	Benzylpenicillin → Phenoxymethylpenicillin
Kragsbjerg, 1995 6	38	4	Left medial breast	*S. pneumoniae*	Negative	*S. pneumoniae*	Cloxacillin → Benzylpenicillin → Phenoxymethylpenicillin
Appalajaru, 2011 7	26	n.a.	Left breast	*S. pneumoniae*	Negative	n.a.	Amoxi‐clavulanic acid + (Draining of abscess) → Linezolid
Miedzybrodzki, 2014 8	35	8	Right lateral breast	*S. pneumoniae* serotype 19A	n.a.	n.a.	Vancomycin → Cefazolin → Cefadroxil

n.d., Not done; n.a., Not available.

It must be noticed that the blood culture was obtained on clinical indication (pyrexia 39.7°C) and would never be dismissed as an insignificant isolate even though the clinical background was rare. At our hospital, blood cultures are obtained by skilled biotechnicians, and we have a low rate of contamination (<2%) most of which are typical skin bacteria.

A Swedish study by Kvist et al. 2. analyzed bacteriological findings in women with lactational mastitis (*n* = 192) and breastmilk donors (*n* = 466). The top ranking bacteria in both groups were CoNS, viridans streptococci, *S. aureus*, group B streptococci, and *E. faecalis*. The strongest association with clinical symptoms was observed for *S. aureus* and group B streptococci. Of special interest to the current study*, S. pneumoniae* was found in 3% of women with mastitis and in 1% of milk donors; this difference was not statistically significant.

A recent Spanish study 3 applied advanced molecular techniques for identification of 105 streptococcal isolates from milk samples delivered by 67 healthy breastfeeding women. None of the isolates were pneumococci, but misidentification may have occurred due to a close genetic relationship between pneumococci and *Streptococcus mitis*.

The relative scarcity of pneumococci in human milk may reflect the presence of a specific bactericidal factor. The factor is a subfraction of α‐lactalbumin with a special configuration maintained by complexing with oleic acid. It remains speculative whether this complex contributes to innate immunity 4.

This case report stresses that *S. pneumoniae* is a rare but possible agent for puerperal mastitis and can be associated with pneumococcal bacteremia. A literature search was consistent with the scarce occurrence of pneumococcal mastitis. Exposure to pneumococci from suckling infants is likely to be common, but the human breast may be protected by a bactericidal complex in the milk.

## Ethics Approval and Consent to Participate

Not applicable.

## Consent to Publish

Written informed consent for publication of the clinical details and/or clinical images was obtained from the patient.

## Availability of Data and Material

Data sharing is not applicable to this article as no datasets were generated or analyzed during the current study.

## Authorship

Both authors contributed to the writing of the manuscript. Both authors read and approved the final manuscript.

## Conflict of Interest

Henrik C. Schønheyder holds a patent: Pneumococcal vaccine and uses thereof; a TLR9 agonist‐adjuvanted pneumococcal vaccine (US 20120052088Al (12)). Sara Vester Hald has no competing interest to declare.
